# Supplementary material for: Genomic data integration by WON-PARAFAC identifies interpretable factors for predicting drug-sensitivity in vivo
Source: Nat Commun. 2019 Nov 6;10:5034. doi: 10.1038/s41467-019-13027-2 (PMC6834616; doi:10.1038/s41467-019-13027-2)
Supplement: Supplementary file 1 — Supplementary Information [file 41467_2019_13027_MOESM1_ESM.pdf]

Supplementary information: Genomic data integration by WON-PARAFAC identifies interpretable factors for predicting drug-sensitivity in vivo

Yongsoo Kim[1,2,3], Tycho Bismeijer[2], Wilbert Zwart[1,4*], Lodewyk FA Wessels[2,5*], Daniel J. Vis[2*]

Keywords Constrained factor analysis; multi-way data integration, drug response prediction

1) Division of Oncogenomics, Oncode Institute, The Netherlands Cancer Institute, The Netherlands
2) Division of Molecular Carcinogenesis, Oncode Institute, The Netherlands Cancer Institute, Amsterdam, The Netherlands
3) Department of Pathology, VU University Medical Center, The Netherlands
4) Department of Biomedical Engineering, Eindhoven University of Technology, The Netherlands
5) Faculty of EEMCS, Delft University of Technology, Delft, The Netherlands

*These authors jointly supervised this work
w.zwart@nki.nl
l.wessels@nki.nl
d.vis@nki.nl

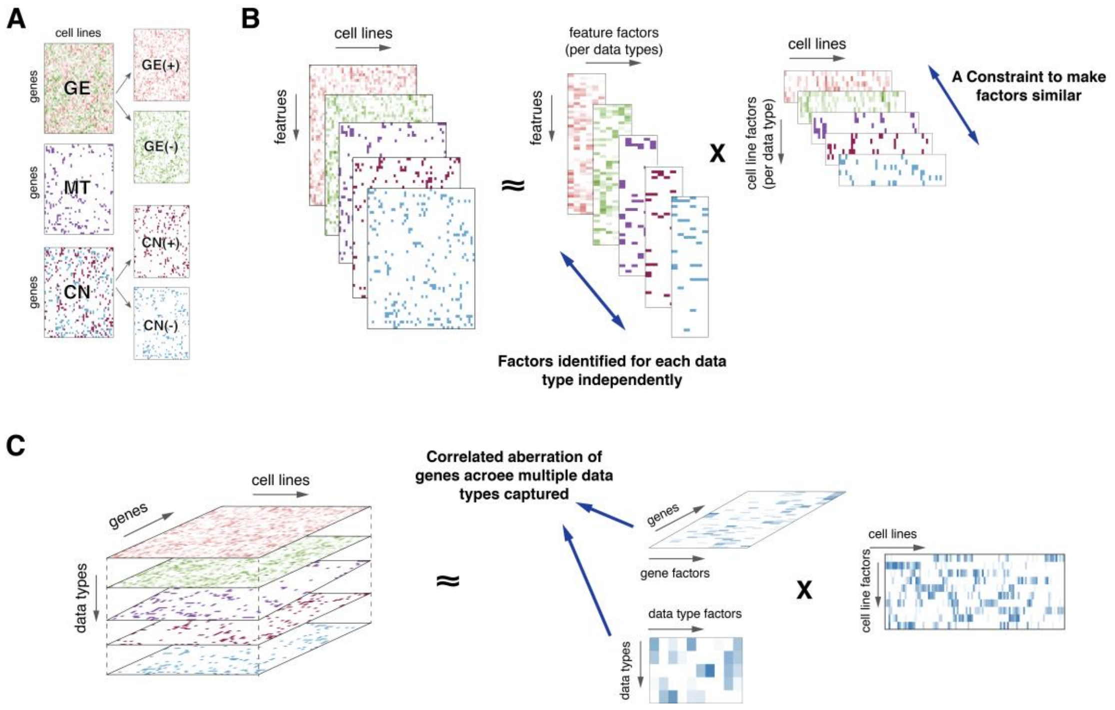

Supplementary Figure 1. Factorization-based approaches for handling multiple genomics data. (A) Multiple genomics data, where positive and negative parts are separated into two separate matrices to ensure non-negativity. (B) Matrix factorization-based approaches (2-way methods) to handle multiple genomics data. (C) A cube-based representation of multiple genomics data (left) and factorization of the cube using parallel factor analysis (PARAFAC; right), a 3-way method.

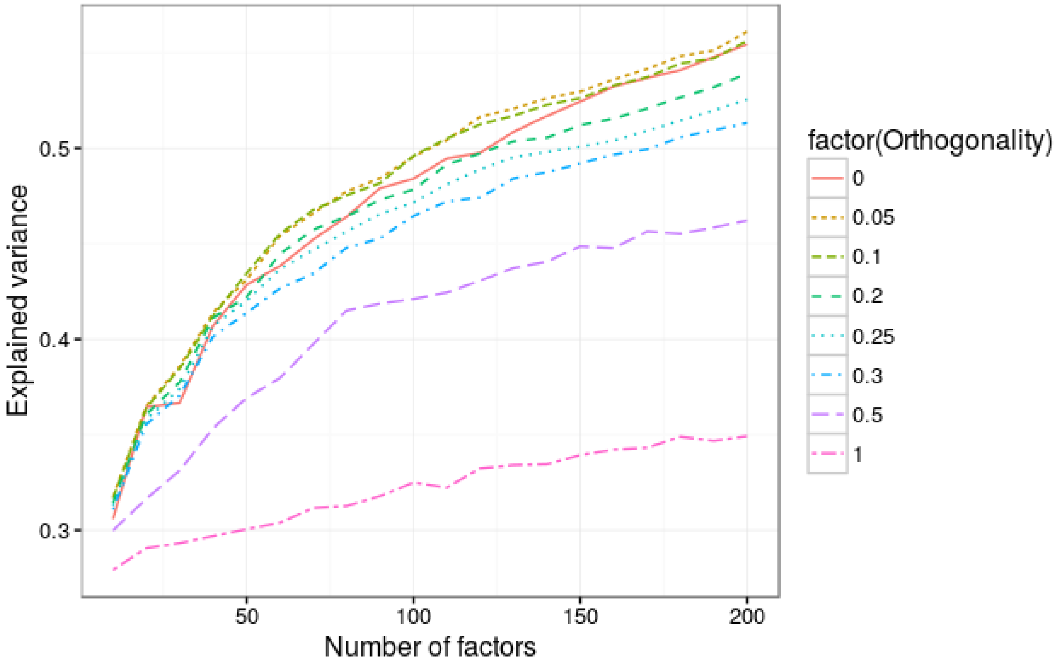

Supplementary Figure 2. Effect of orthogonality constraint on WON-PARAFAC with a diverse number of factors. Models with different level of orthogonality constraints (1 is the highest, and 0 is no constraint) are indicated with different line types and colors.

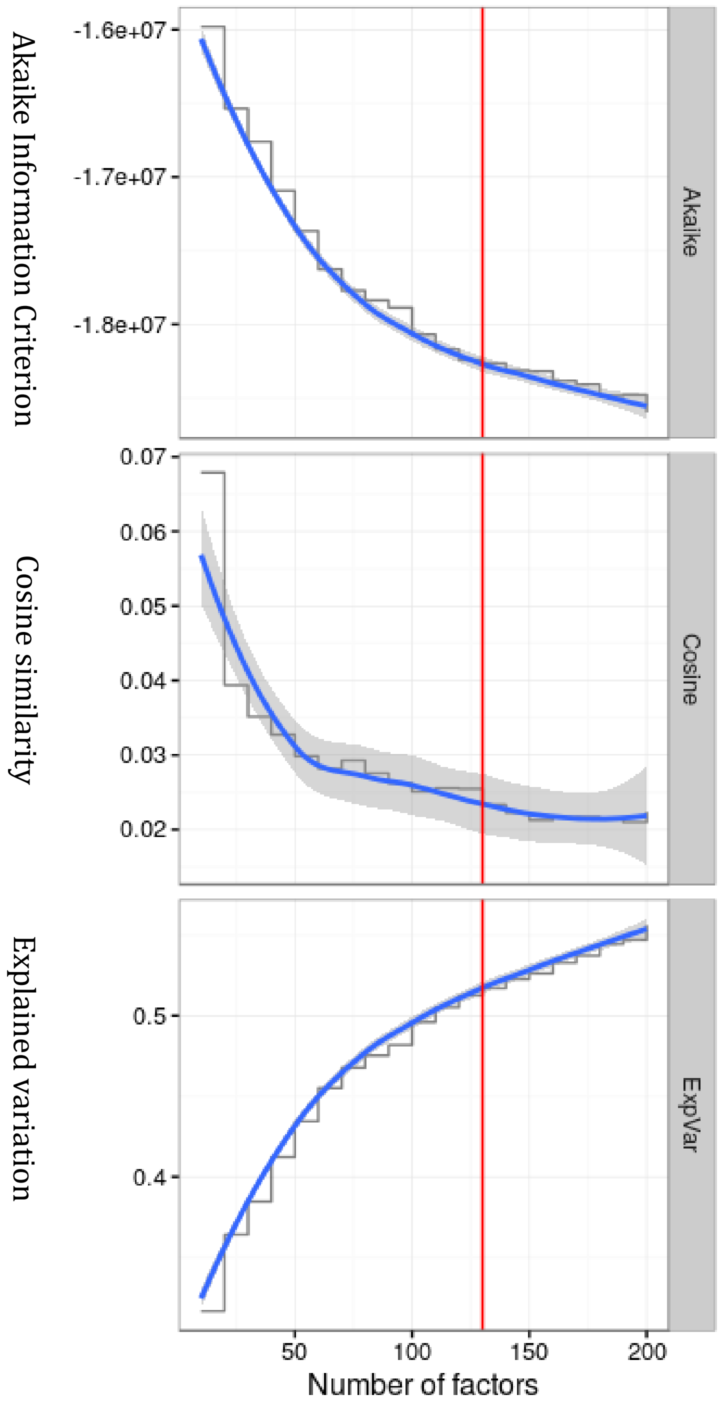

Supplementary Figure 3. AIC, cosine similarity and explained variation of WON-PARAFAC with a diverse number of factors. Actual measures and smoothed profiles are in gray and blue, respectively. The red vertical line indicates final choice, 130 factors.

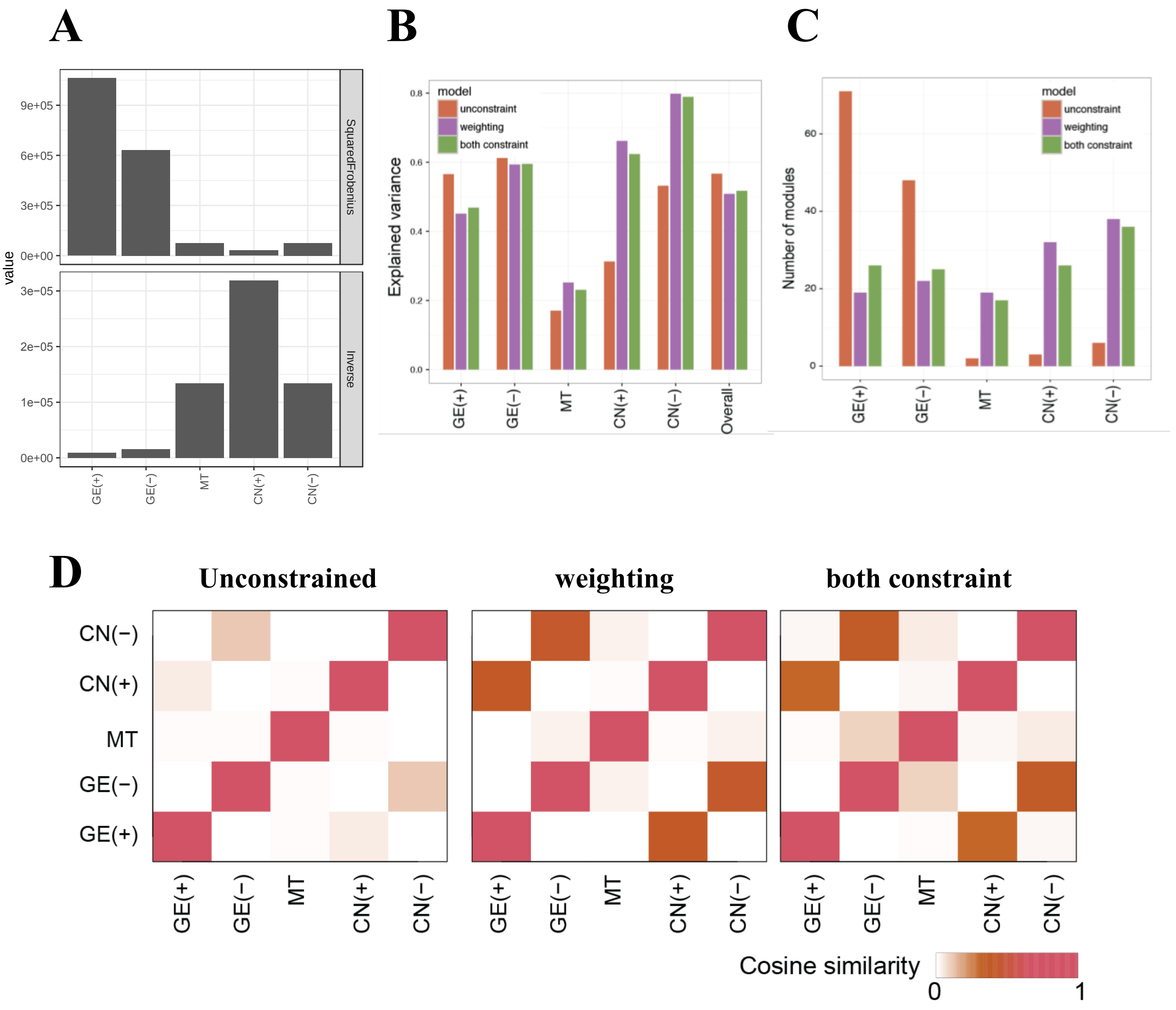

Supplementary Figure 4. (A) Bar plots showing square Frobenius norm (top) and it's inverse (bottom). (B and C) Bar plots comparing WON-PARAFAC (green bar) with non-negative PARAFAC (orange) and non-negative PARAFAC with weighting scheme (purple) in terms of explained variations and number of factors to which each of the data types contributes the most. (D) Heatmaps comparing the three methods in terms of identifying shared pattern across the data types measured by cosine similarity.

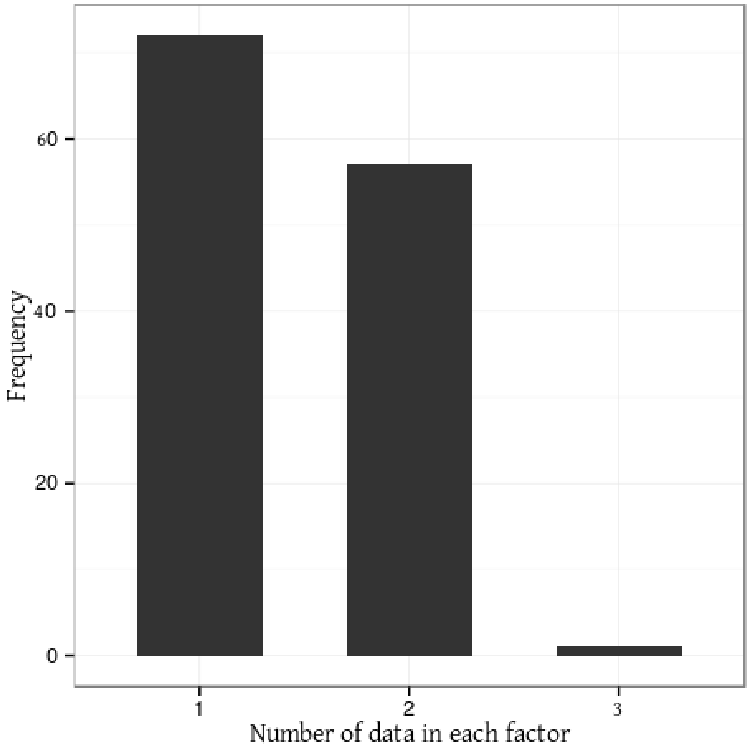

Supplementary Figure 5. A bar plot indicating frequencies of the number of data types involved among 130 factors. Given a factor, data types with loadings > 20% of the largest loading are considered actively involved for the factor.

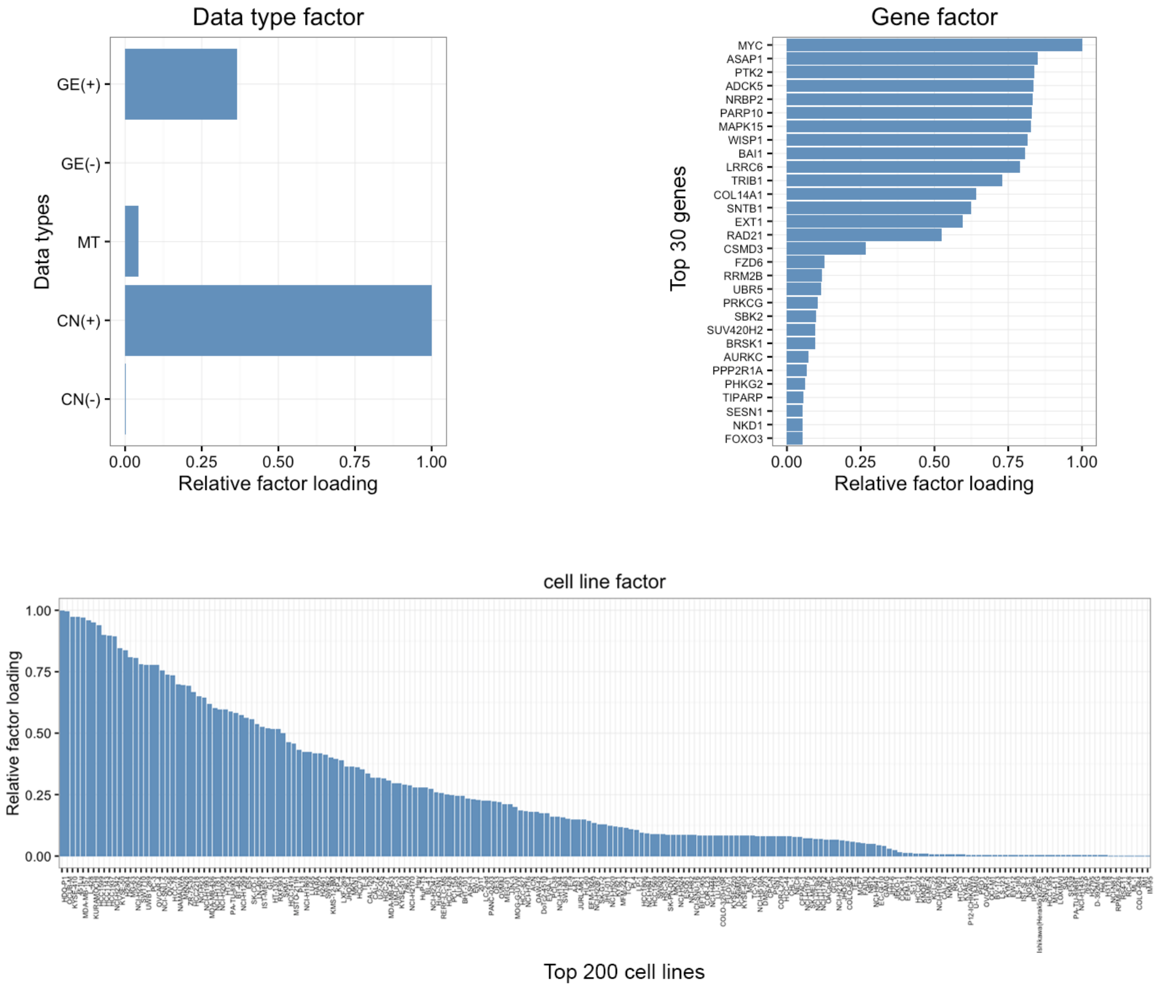

Supplementary Figure 6. Sorted bar plots showing 94th-factor loadings of top 30 genes (top right), data types (top left) and top 200 cell lines (bottom).

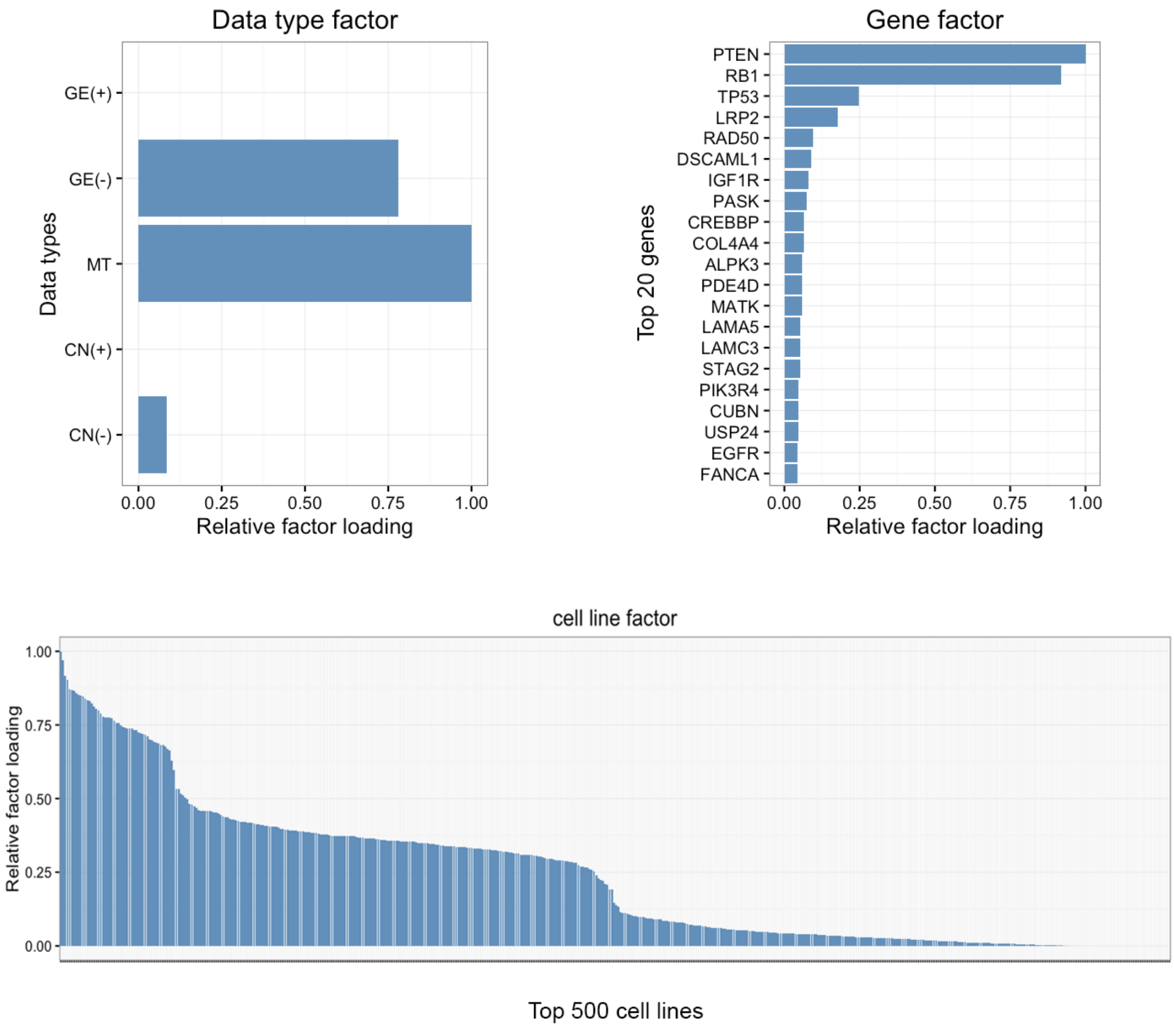

Supplementary Figure 7. Sorted bar plots showing 58th-factor loadings of top 20 genes (top right), data types (top left) and top 500 cell lines (bottom).

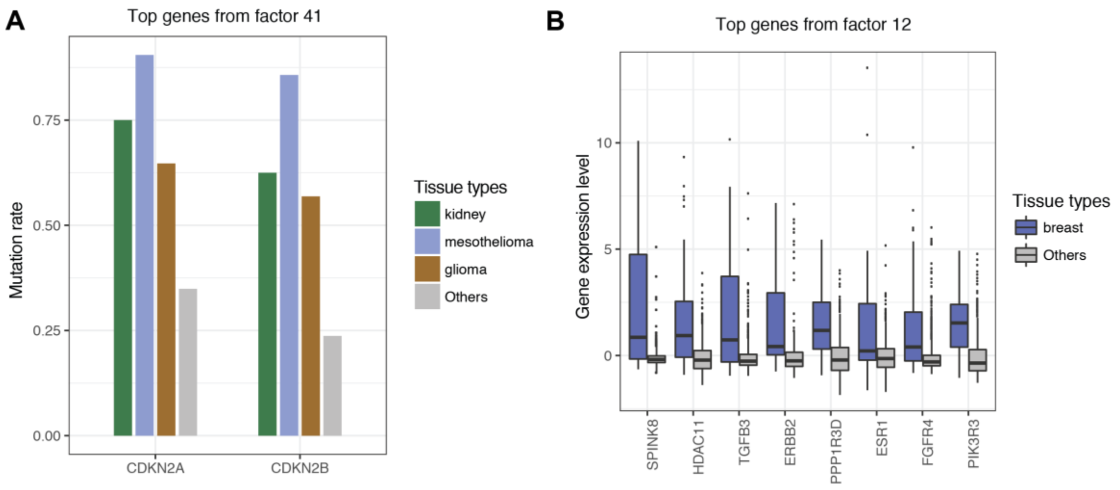

Supplementary Figure 8. Alterations in top genes and their alterations in enriched tissue types for 41st factor (A) and 12th factor (B). Different colors are used to indicate different tissue types.

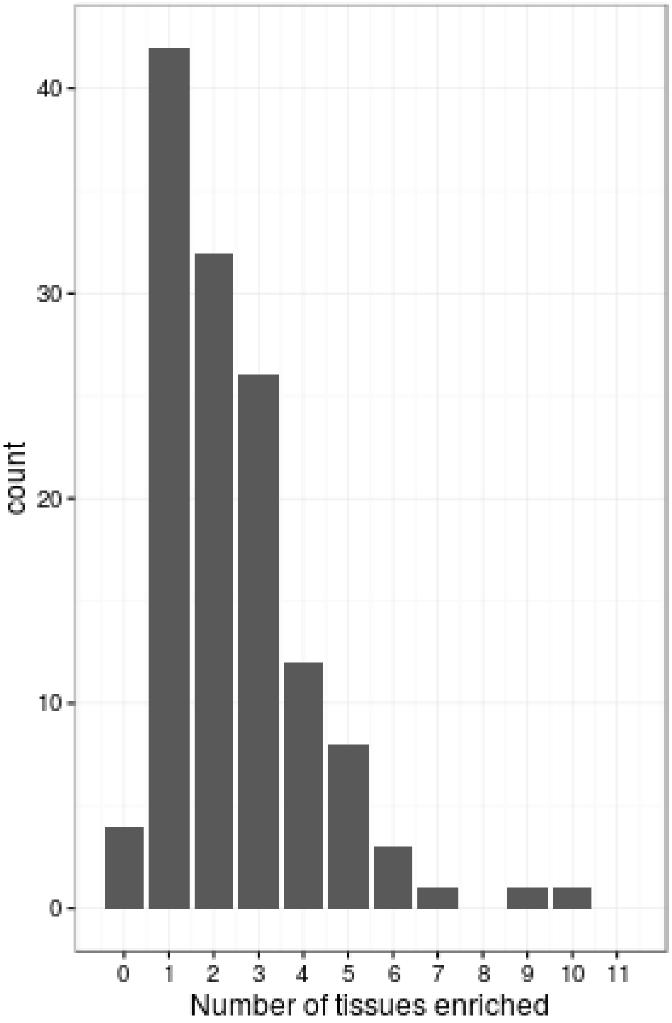

Supplementary Figure 9. Histogram showing the frequency of the number of tissues enriched per factor in CSEA.

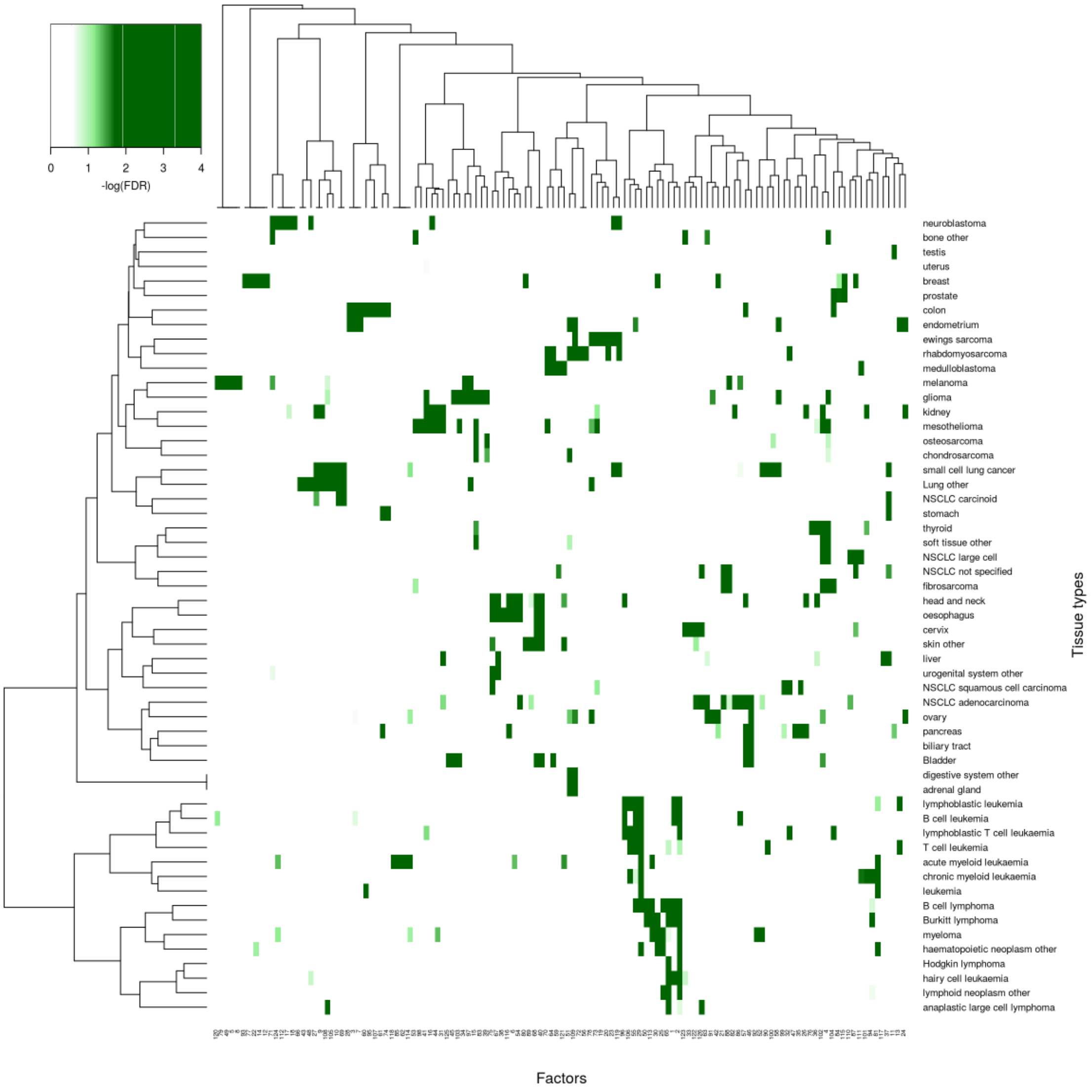

Supplementary Figure 10. A heat map shows the association between tissue types and factors. The color gradient indicates significance score (negative log converted false discovery rate).

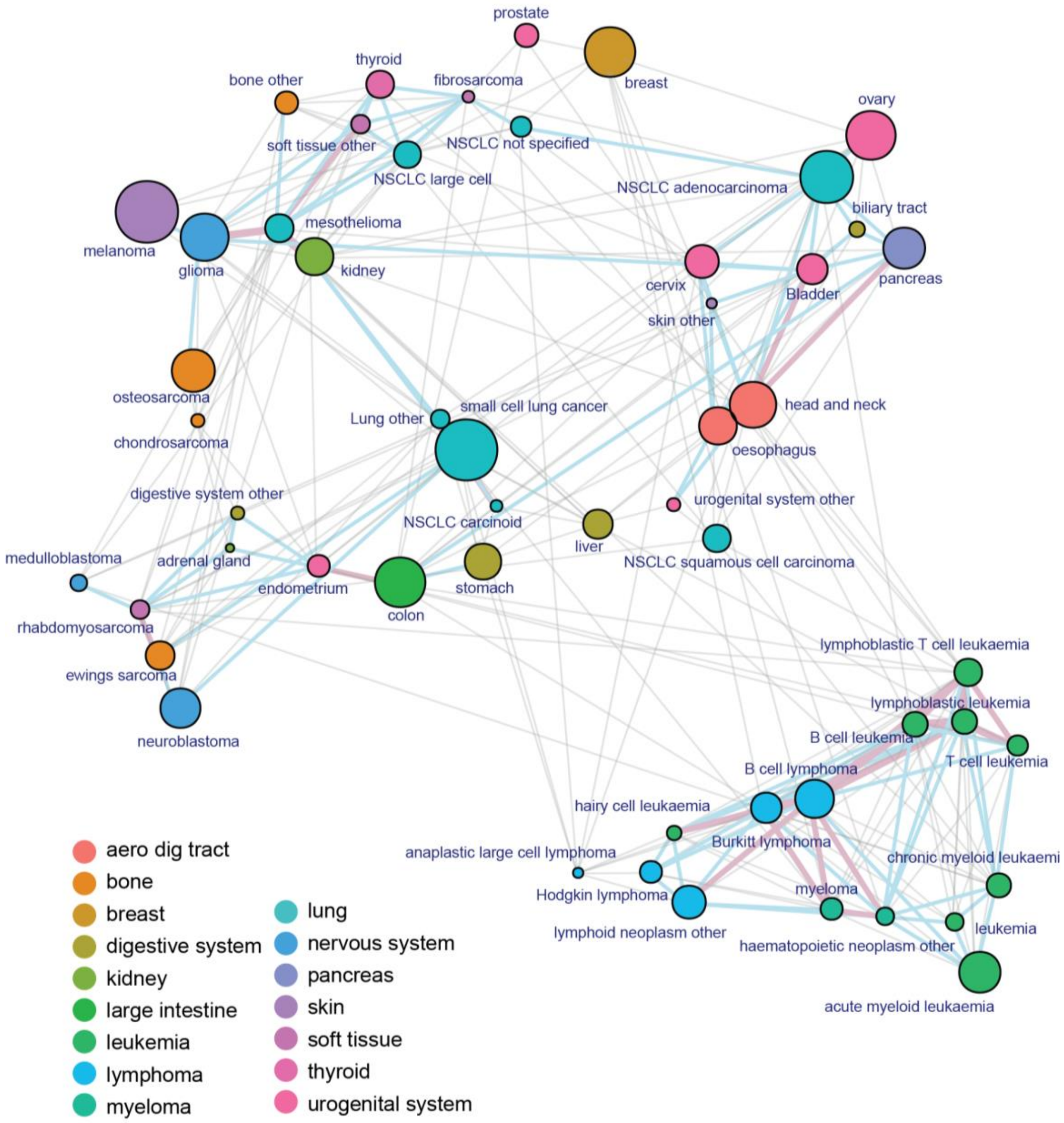

Supplementary Figure 11. Relationships between tissue types represented in a network where nodes and edges represent tissue types and the presence of shared factors, respectively. The node positions are determined by t-SNE that preserves Jaccard distance between tissue types measured on binary factor-tissue type association matrix (FDR<0.2). Broader tissue type classification is indicated by node color. The number of shared factors is indicated by gray (1 factor), blue (2 factors) and pink (more than equal to 3 factors) edges.

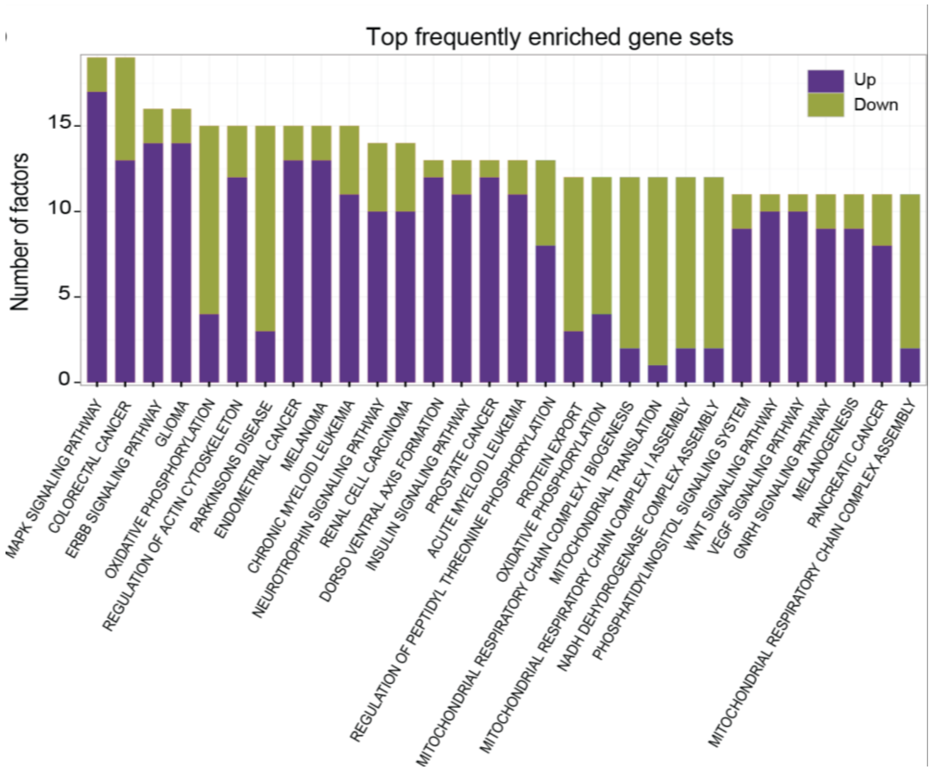

Supplementary Figure 12. Top 30 frequently enriched gene sets among KEGG pathways, biological processes, and hallmarks across 130 factors. Up and down-regulation are indicated by purple and dark green bar, respectively.

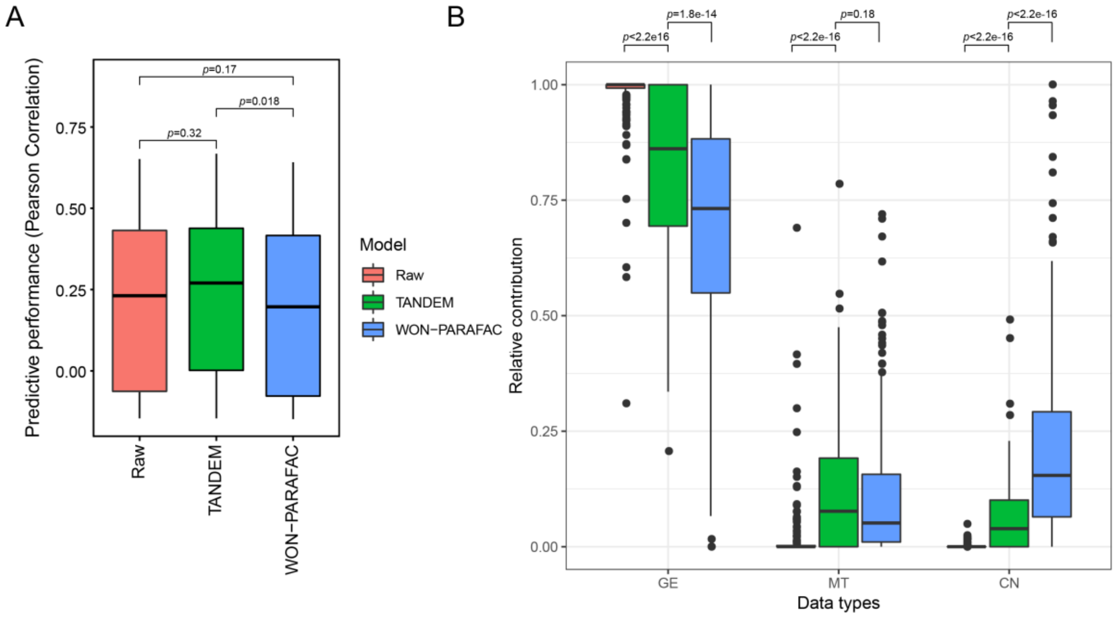

Supplementary Figure 13. Comparison of the raw feature-based EN (raw; red), TANDEM (TANDEM; green) and the factor-based EN (WON-PARAFAC; blue) in the prediction of drug response. (A) Comparison of the predictive performance of the three methods. Standard notations are used for elements of the boxplot (i.e. upper/lower hinges: 75th/25th percentiles; inner-segment: median; and upper/lower whiskers: extension of the hinges to the largest/smallest value at most 1.5 times of interquartile range). The *p*-values from the t-test are indicated at the top. (B) Relative contributions of gene expression (GE), mutation (MT), and copy number data (CN) in prediction of 256 compound responses.

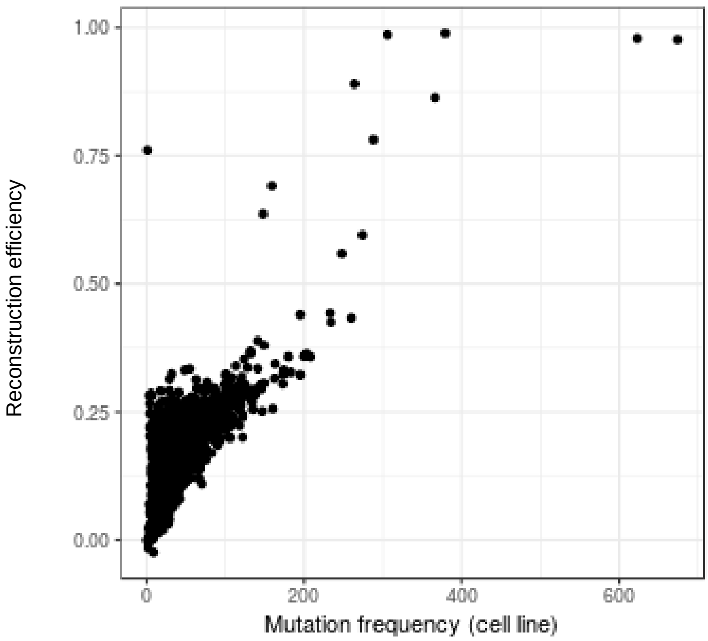

Supplementary Figure 14. A scatter plot compares mutation frequency (number of cell lines; x-axis) and the reconstruction efficiency by the WON-PARAFAC factors (explained variation; y-axis) per gene.

A

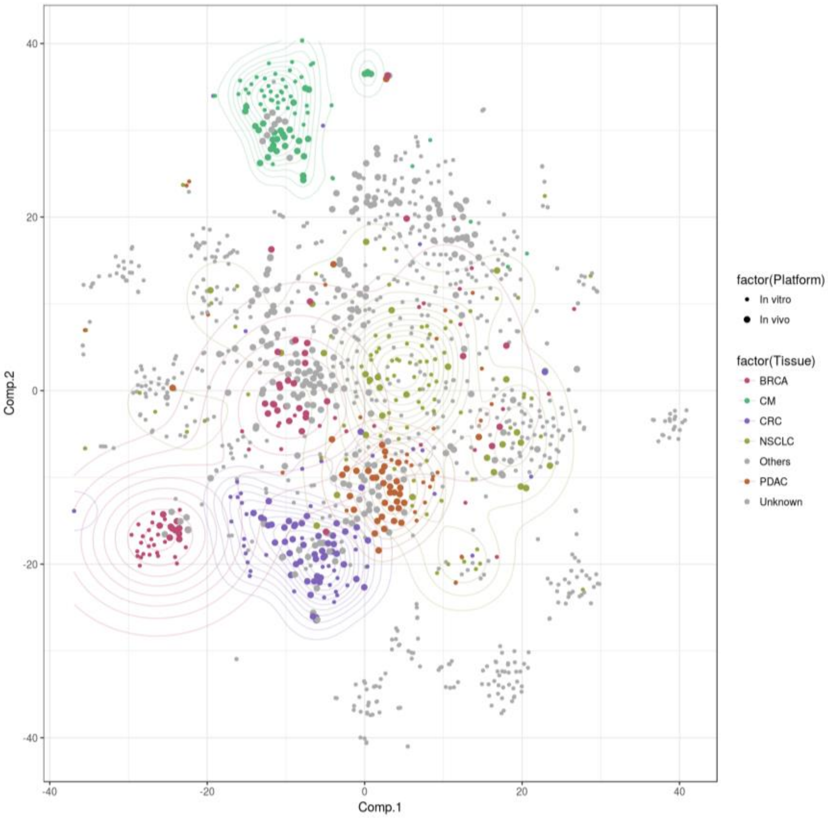

B

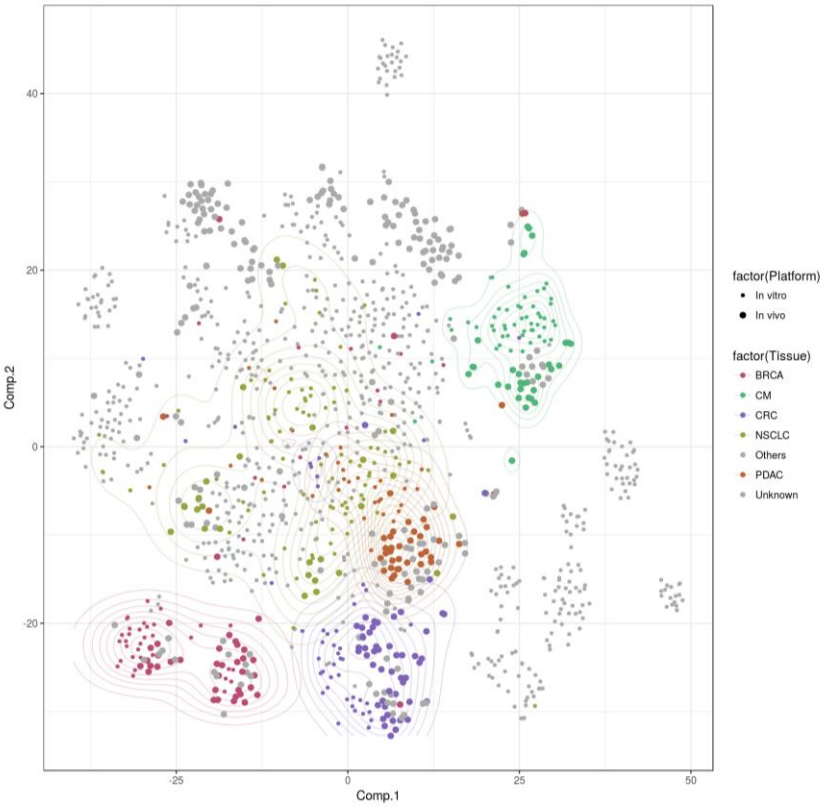

Supplementary Figure 15. *t*-SNE plots of cell lines and PDXs using factors (A) and raw features (B). Tissue types and type of model are indicated by node color and size, respectively.

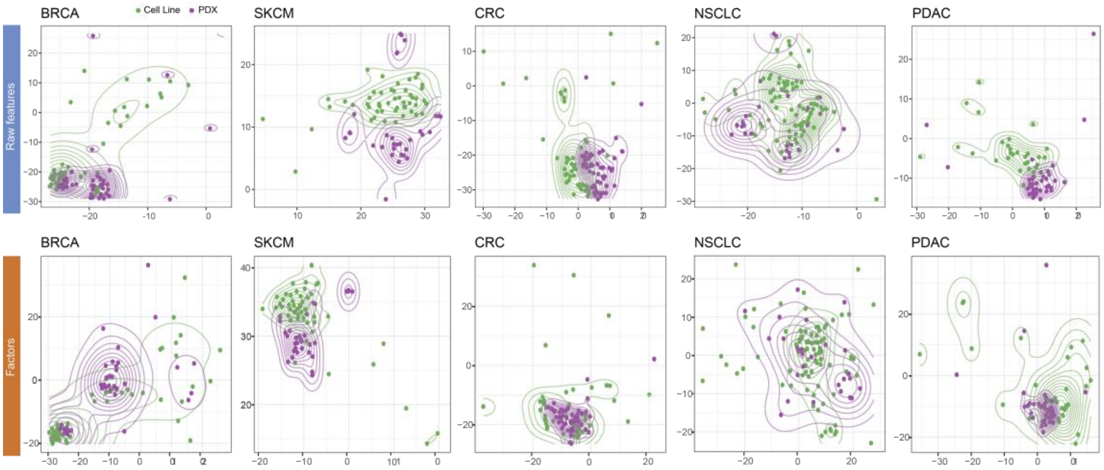

Supplementary Figure 16. *t*-SNE plots of cell lines and PDXs using raw features (top) and factors (bottom), separated by tissue types. Type of model is indicated by node color.

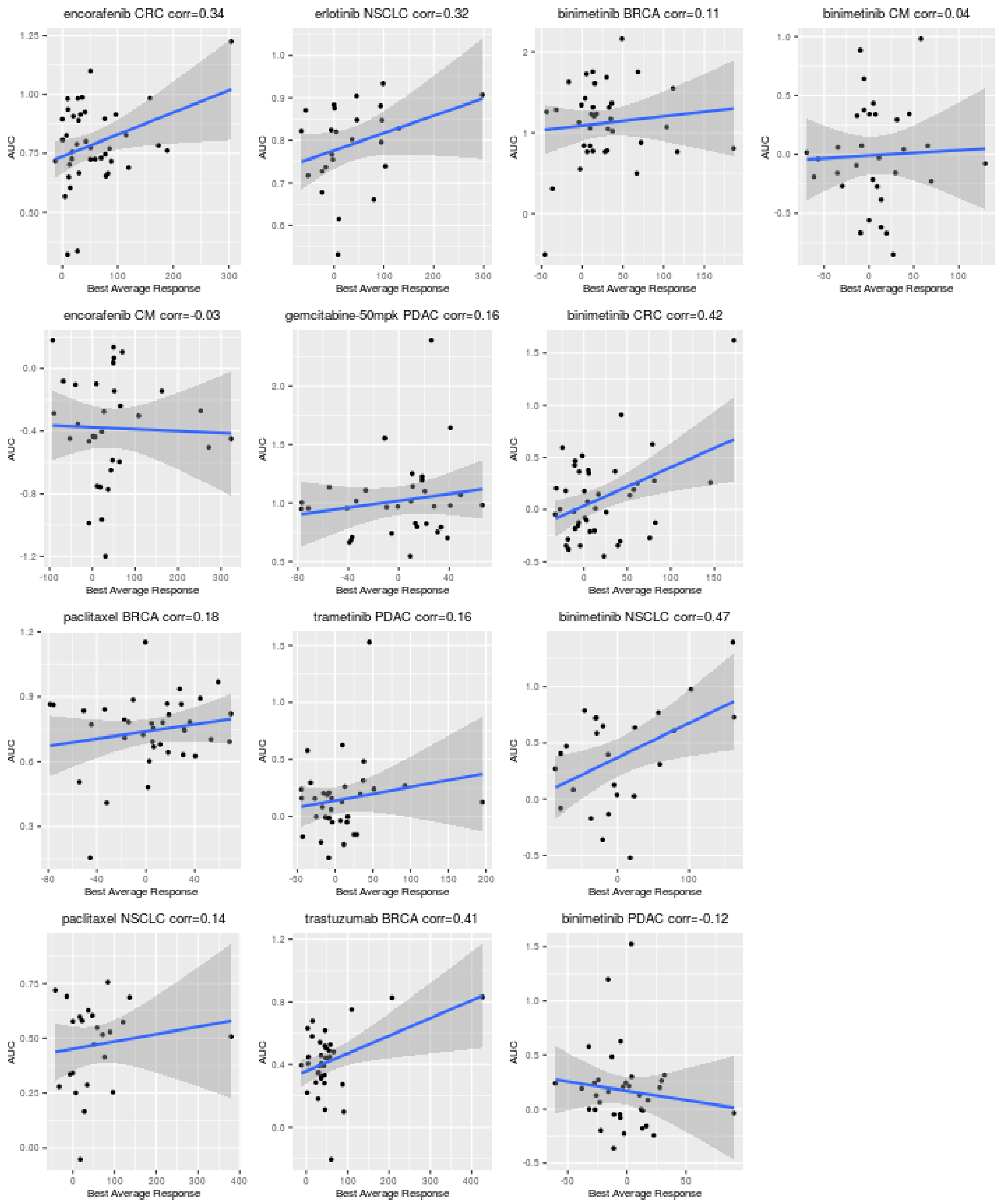

Supplementary Figure 17. Scatterplots compare measured and predicted drug responses of PDXs using ENs on compressed features. Drug name, tissue type and Pearson correlation are indicated at the top of each panel. Linear regression and 95% confidence intervals are denoted by blue line and shadow.

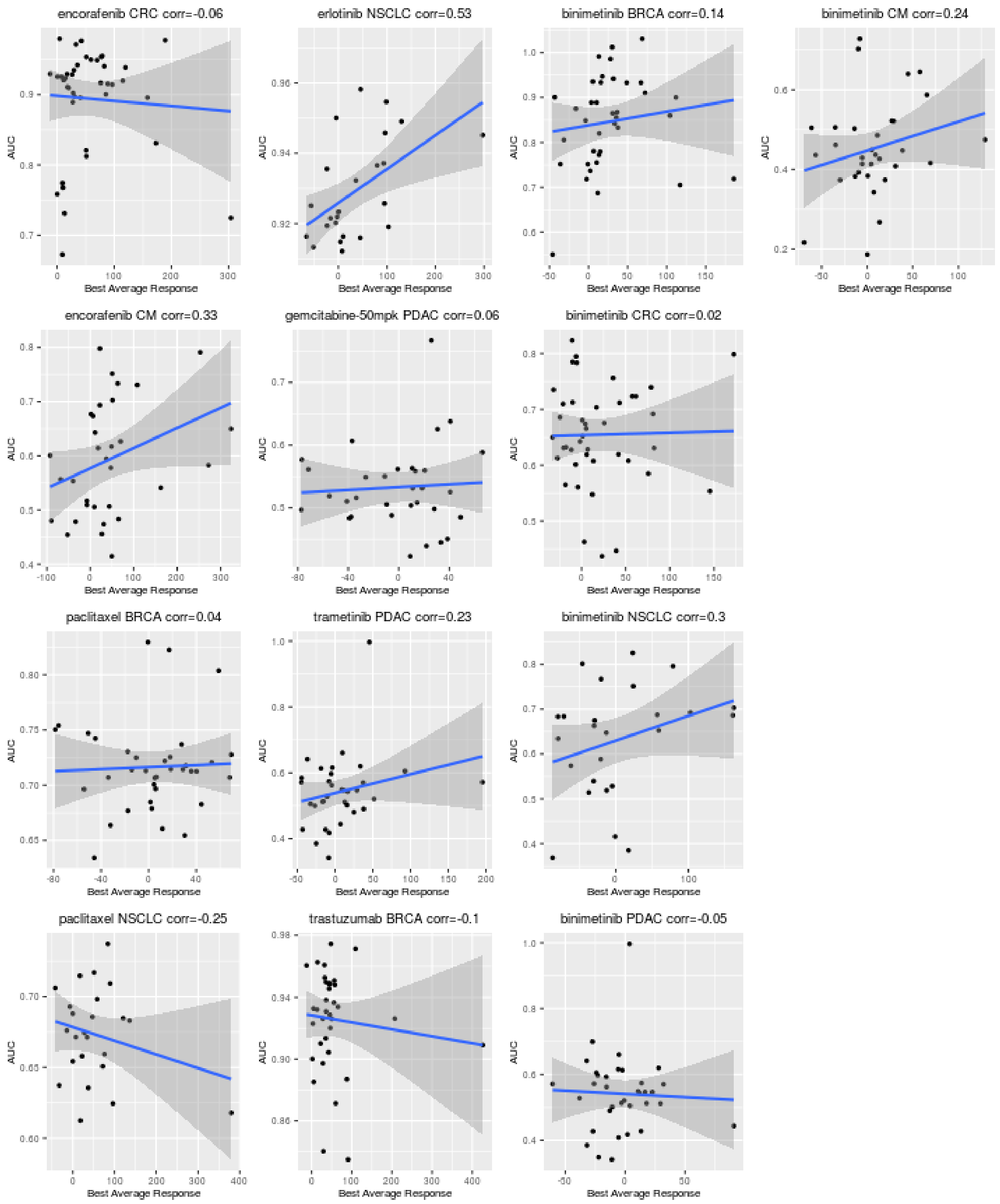

Supplementary Figure 18. Scatterplots compare measured and predicted drug responses of PDXs using ENs on raw features. Drug name, tissue type and Pearson correlation are indicated at the top of the panel. Linear regression and 95% confidence intervals are denoted by blue line and shadow.

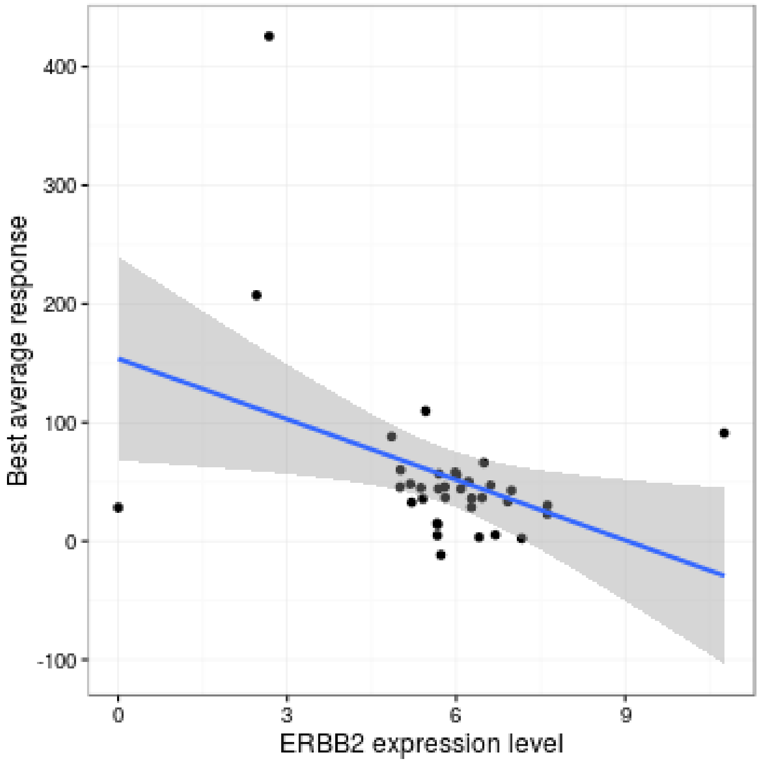

Supplementary Figure 19. Scatterplots compare the best average response to trastuzumab and ERBB2 expression levels of PDXs. Linear regression and 95% confidence intervals are denoted by blue line and shadow.

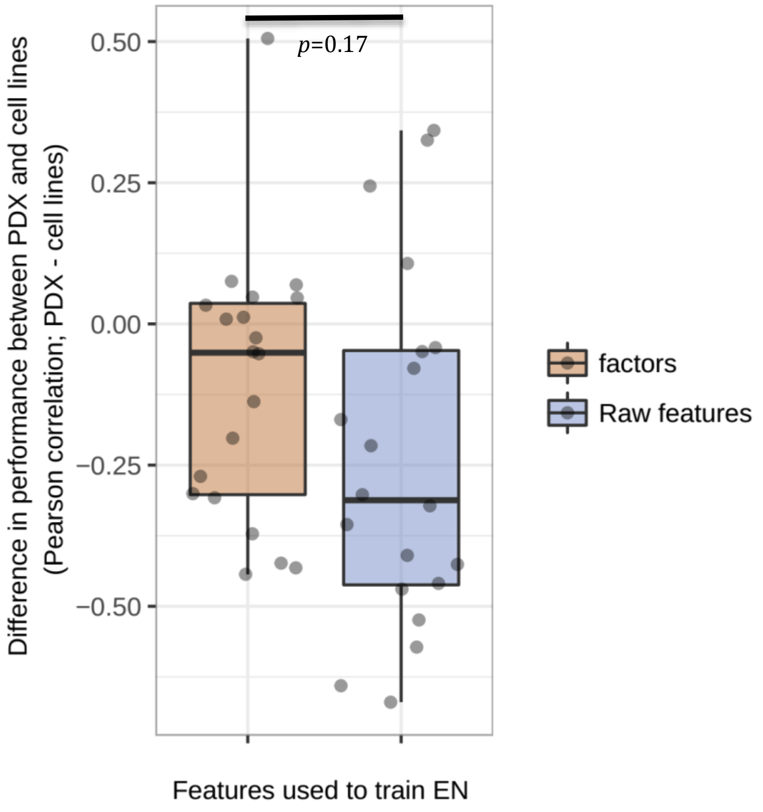

Supplementary Figure 20. Boxplots showing performance difference of ENs in cell lines and PDXs. The Pearson correlation measured in PDX is subtracted by that in cell lines. Features used for training ENs is indicated by box colors. P-value from a t-test comparing fold change values is indicated above.
