# Supplementary material for: Genomic data integration by WON-PARAFAC identifies interpretable factors for predicting drug-sensitivity in vivo
Source: Nat Commun. 2019 Nov 6;10:5034. doi: 10.1038/s41467-019-13027-2 (PMC6834616; doi:10.1038/s41467-019-13027-2)
Supplement: Supplementary file 3 — Description of Additional Supplementary Files [file 41467_2019_13027_MOESM3_ESM.pdf]

Description of Additional Supplementary Files

File Name: Supplementary Data 1
Description: 130 factors derived from pan-cancer cell lines and their associations to tissue type, pathways, and drug response. Data file includes factor loadings of PDXs from PDXE.
